# Ag–Se/Nylon Nanocomposites Grown by Template-Engaged Reaction: Microstructures, Composition, and Optical Properties

**DOI:** 10.3390/nano12152584

**Published:** 2022-07-27

**Authors:** Valentina Krylova, Nijolė Dukštienė, Henrieta Markevičiūtė

**Affiliations:** Department of Physical and Inorganic Chemistry, Faculty of Chemical Technology, Kaunas University of Technology, Radvilėnų Str. 19, 50254 Kaunas, Lithuania; nijole.dukstiene@ktu.lt (N.D.); henrieta.markeviciute@ktu.edu (H.M.)

**Keywords:** Ag_2_Se, nylon 6, flexible inorganic-organic composite, ProX-SEM-EDS, optical properties

## Abstract

Ag–Se nanostructure films were deposited on a–Se/nylon templates by a template-engaged reaction. Firstly, amorphous selenium (a–Se) was deposited on nylon by employing the chemical bath deposition method while using H_2_SeO_3_ and Na_2_SO_3_ solutions with an increasing selenium deposition time. Then, these a–Se/nylon templates were exposed into AgNO_3_ solution at ambient temperature and pressure. The Ag–Se/nylon nanocomposites surface morphology, elemental and phase composition, and optical properties were monitored depending on the selenium deposition time on nylon. Scanning electron microscopy (SEM) analysis confirmed the development of a very complex surface composed of pyramidal-like sub-micron structures, agglomerates, and grid-like structures. Energy dispersive spectroscopy (EDS) proved the presence of carbon, oxygen, nitrogen, selenium, and silver. SEM/EDS cross-sectional analysis confirmed the multilayer character with different individual elemental composition in each film layer. X-ray diffraction analysis revealed a polycrystalline Ag_2_Se phase with or without metallic Ag. The RMS value obtained from atomic force microscopy varies from 25.82 nm to 57.04 nm. From the UV-Vis spectrophotometry, the direct optical band gaps were found to be 1.68–1.86 eV. Ag–Se/nylon composites exhibit high refractive indices in the near infrared region.

## 1. Introduction

Currently, a lot of attention is devoted to the flexible photovoltaic membranes, as they can maintain the required durability and fulfil the aesthetic, building-physics requirement(s) [1]. Therefore, the demand for a sustainable and semi-permanent energy-harvesting system, which converts solar energy to electricity, has been continuously increasing. Photovoltaic devices also need to be mechanically flexible to be employed as an energy supplier for a curved electronic device. Hybrid organic–inorganic composites are considered as very attractive and promising materials due to the diverse properties and additional functionalities as compared with those of individual components [2]. Thin films of solar light absorbers such as metal chalcogenides are of extraordinary interest for the production of solar selective coatings, large area arrays, and photovoltaic cells. The modification of flexible polymers with metal chalcogenides not only allows control an architecture of resultant solar absorbers, but also enables the creation of flexible materials with the unique microstructures and optical properties. Printing and coating methods are two main techniques to deposit thin films onto the flexible polymer surface [1]. Recently, surface modification of organic polymers with metal chalcogenides thin films via different coating techniques have been reported [3].

Owning to its high mechanical strength and high chemical and thermal stability, nylon 6 (polyamide 6) is a promising candidate for high-performance flexible energy-harvesting systems. Among a variety of metal chalcogenides, silver selenide displays many interesting properties. Ag_2_Se exists in two polymorphs at atmospheric pressure: a low-temperature orthorhombic phase (*α*-Ag_2_Se), and a high-temperature cubic phase (*β*-Ag_2_Se) [4]. *β*-Ag_2_Se is distinguished for its Seebeck coefficient (−150 μ VK^−1^ at 300 K [5]), large magnetoresistance [6], and excellent thermoelectric properties [7]. The Ag_2_Se nanowires phase nature greatly influences electrical conductivity [8]. Authors [9] stated a super-ionic α-Ag_2_Se conductor was employed in photo chargeable batteries. Environmentally friendly, an n-type nanocrystalline, Ag_2_Se thin film exhibits the direct bandgap of 1.8 eV [10].

From the broad list of available literature, only a few articles have reported that a facile strategy has been developed to prepare flexible Ag_2_Se films and Ag_2_Se-based composite films on nylon [7,11,12]. These multiscale Ag_2_Se nanoparticles with or without Ag nanoparticles on nylon exhibit a high power factor and excellent flexibility [7,12]. Through literature analysis, we did not succeed in finding any publication devoted to optical properties of Ag_2_Se/nylon composites. Considering the rapid application of hybrid materials in opto-electronic modules, such study remains relevant, significant, and timely.

Template-assisted synthesis represents a straightforward, adaptable, and successful nanomaterial synthesis approach. In this approach, the templates may serve as physical scaffolds, against which other materials are assembled, or templates are engaged in synthesis as one of the reactants (template-engaged reactions). Recently, several teams have described the synthesis of Ag_2_Se nanotubes [13,14], nanowires [5,15], and Se/Ag_2_Se/core/shell nanocables [14] by this technique. The authors emphasised that the resulting Ag_2_Se have retained the both shape and morphology of trigonal Se template with good precision.

In this study, compact Ag–Se/nylon semiconductor nanocomposites were formed via a template-engaged reaction, which could convert the amorphous selenium (a–Se) layer on nylon into an Ag–Se film. The sequential deposition method was explored. The type of the template, as well as its amount, exerts a strong influence on the structure and the properties of the resulting composites. Firstly, a–Se was deposited on nylon 6 while using the chemical bath deposition (CBD) method by mixing solutions of H_2_SeO_3_ and Na_2_SO_3_ and changing the time of selenium deposition. These a–Se/nylon templates were then exposed to the AgNO_3_ solution. The Ag–Se/nylon nanocomposites surface morphology, elemental and phase composition, and optical properties were monitored depending on the selenium deposition time on nylon. The results were interpreted, discussed, and compared with some of the currently available state-of-the-art reports.

## 2. Materials and Methods

### 2.1. *Polymer*

The thermoplastic matrix which we used was semi crystalline nylon 6 *Tecamid 6*, (hereinafter referred as ‘nylon’) produced by Ensinger GmbH (Germany). The 500 μm-thick nylon film was opaque. The density was 1.13 g/cm^3^, moisture absorption 3%, water absorption to equilibrium 9.5%. The experiments were performed on strips of 2′ 6 cm^2^ in size. Prior to the experiments, nylon film cuts were washed with Na_2_CO_3_, and afterward treated in distilled water at 100 °C for 2 h. The criterion for the quality treatment of the nylon substrate surface was its uniform wetting with distilled water. After treatment, the substrates were stored in a desiccator.

### 2.2. *Chemicals*

The distilled water and as received analytical grade reagents were used to prepare freshly solutions for each experiment. Selenous acid (H_2_SeO_3_, 99.0%) and sodium sulphite heptahydrate (Na_2_SO_3_∙7H_2_O, 99.0%) were obtained from Reachim, Russia. Sulphur acid (H_2_SO_4_, 96.0%), silver nitrate (AgNO_3_, 99.0%), and sodium carbonate hydrate (Na_2_CO_3_·10H_2_O, ≥99.0% (calc. based on dry substance)) were purchased from Aldrich Chemical Co.

### 2.3. Sample Preparation and Theoretical Background

The first step involved the formation of a selenium film via the CBD method. The concentrations of H_2_SeO_3_ and Na_2_SO_3_ solutions and temperature that yielded a superior a–Se/nylon template, with respect to continuity, smoothness, and the adherence of the selenium film to the substrate, were chosen for further experiments. For Se film deposition, nylon strips were exposed in 0.1 M H_2_SeO_3_ and 0.15 M Na_2_SO_3_ solution (pH 2 adjusted with H_2_SO_4_) for 6, 12, 24, and 30 h at 20 ± 1 °C. The red amorphous selenium (a–Se) isolated according to the following equation [16,17]:

H_2_SeO_3__(aq)_ + 2Na_2_SO_3__(aq)_ → Se_(s)_ + 2Na_2_SO_4__(aq)_ + H_2_O. (1)

The half reactions for the reduction and oxidation of selenous acid and the sulphite ion, respectively, are the following Equations (2) and (3):

H_2_SeO_3__(aq)_ + 4H^+^_(aq)_ + 4e^−^ → Se_(s)_ + 3H_2_O, (2)

SO_3_^2^^−^_(aq)_ + H_2_O − 2e^−^ → SO_4_^2^^−^_(aq)_ + 2H^+^_(aq)_. (3)

The reduction potentials (*E*^0^_red_) are 0.74 V for Equation (2) and 0.17 V for Equation (3), respectively. Therefore, sodium sulphite reduces selenite readily, and the redox reaction is spontaneous [16].

For Equation (1), an acidic environment and the stoichiometry of the initial reacting materials are essential in order to prevent the formation of polythionates. Monoselenotrithionic acid forms with an excess of H_2_SO_3_ solution are added to the solution of H_2_SeO_3_ with ratio 3:1 [17]:

H_2_SeO_3(aq)_ + 3H_2_SO_3(aq)_ → H_2_SeS_2_O_6(aq)_ + H_2_SO_4(aq)_ + 2H_2_O. (4)

Diselenotetrathionic acid forms when H_2_SO_3_ solution is added to an excess of H_2_SeO_3_ [17]:

2H_2_SeO_3(aq)_ + 5H_2_SO_3(aq)_ → H_2_Se_2_S_2_O_6(aq)_ + 3H_2_SO_4(aq)_ + 3H_2_O. (5)

In an acidic solution, polythionic acids decompose with the release of elemental selenium. Selenotritithionic acid H_2_SeS_2_O_6_ is more stable than diselenothionic acid H_2_Se_2_SO_6_, which decomposes with the release of elemental selenium at room temperature [17].

Afterward the selenium deposition, the a–Se/nylon samples were cleaned with C_2_H_5_OH to remove a poorly adhering film. Then a–Se/nylon samples were thoroughly washed with hot distilled water, and, between the subsequent processing steps, were stored in a desiccator. During the second stage, these a–Se/nylon templates were treated for 2 h with 0.1 M solution of AgNO_3_ (pH 6.35) at 20 ± 1 °C temperature in a thermostatic vessel.

The solid phase formation at the template/solution interface is a dynamic non-equilibrium process requiring careful consideration of the physicochemical pathways by which they proceed. The solubility of the starting material is the determining indicator for the thermodynamic reaction feasibility in thin films deposition. The change in system Gibbs free energy is also determinant in the solid-state reaction pathway [18,19]. Additionally, the ion-exchange reactions can precede through simple mutual diffusion [20]. The highly reactive elemental Se is an excellent template for the synthesis of various metal selenides even at ambient temperature [5]. If wet films of silver and selenium are stocked together, the reaction–diffusion process starts to yield the silver selenide [21,22]. The formation of Ag_2_Se phases in the Ag–Se/nylon nanocomposite could be explained through complex mechanism reactions. When refluxed in an aqueous medium containing Ag^+^ cations, amorphous selenium disproportionates into Se^2^^−^ and SeO_3_^2^^−^ anions:

3Se_(s)_ + 3H_2_O → 2Se^2^^−^_(ad)_ + H_2_SeO_3__(ad)_ + 4H^+^_(aq)_. (6)

Ag^+^ ions react with adsorbed chalcogenides particles (Se^2^^−^, SeO_3_^2^^−^) to generate insoluble nanoparticles, which are in situ deposited on the a–Se/nylon template to produce Ag–Se/nylon nanocomposites. The major reaction describing Ag_2_Se formation can be written as follows [5]:

3Se_(s)_ + 6AgNO_3(aq)_ + 3H_2_O → 2Ag_2_Se_(s)_ + Ag_2_SeO_3(s)_ + 6HNO_3__(aq)_. (7)

As the hydrophilic nylon is treated in the acidified selenium precursor solution, SO_3_^2–^, SO_4_^2–^, and SeO_3_^2–^ ions can diffuse into the sub-surface space of nylon, and potentially bind to the charged sites of nylon, such as the ionised functional groups −CONH and –NH [23]. Ag^+^ cations could diffuse into the a–Se/nylon template, and the formation of a sub-product within the template matrix—such as Ag_2_SO_4_, Ag_2_SO_3_, and Ag_2_SeO_3_—is probable.

The formed Ag–Se/nylon nanocomposites were thoroughly rinsed with hot distilled water, dried and stored in the desiccator over CaCl_2_. Throughout the text, the obtained nanocomposites were labelled as Ag–Se-6/nylon, Ag–Se-12/nylon, Ag–Se-24/nylon and Ag–Se-30/nylon, where the added number refers to the selenium deposition time.

### 2.4. Testing Procedures

The solution pH was measured by using a pH-meter WTW330 (Xylem Analytics Germany Sales GmbH & Co. KG WTW, Weilheim, Germany). An optical microscope CX31 equipped with a C-5050 photo camera (Olympus Corporation, Tokyo, Japan) was used to take the images of uncoated nylon and the obtained Ag–Se/nylon composites. The X-ray diffraction (XRD) analysis was performed on a Bruker Advance D8 diffractometer with Bruker LynxEye counting detector. The operating parameters were the tube voltage of 40 kV, and the tube (emission) current of 40 mA. A Ni 0.02 mm filter selected CuKα (λ = 0.154178 nm) radiation. XRD patterns collected 2θ = 30–70° at a scanning rate of 1° min^−1^ by using the coupled two theta/theta scan type. Scanning electron microscopy coupled with energy-dispersive X-ray spectroscopy (EDS/SEM) analyses were conducted using a Phenom ProX desktop scanning electron microscope (LOT-QuantumDesign) with a high sensitivity multi-mode backscatter electron (BSE) detector. Resolution was ≤8 nm. Primary-beam energy was 0.15 kV EHT. Atomic force a NanoWizard^®^3 NanoScience microscope (JPK Instruments, Bruker Nano GmbH, Berlin, Germany) with pyramidal-shaped i-type silicon cantilever (0.01–0.025 ohm/cm, spring constant of 2 N/m) operated in the contact mode. The AFM images scanning area was 30 × 30 μm^2^. Topographical parameters were evaluated using JPKSPM Data Processing software (Version spm-4.3.13). The diffuse reflectance spectra of the composites were recorded by using a UV-Vis spectrophotometer Lambda 35 within the range 380–1100 nm. The reflectance data were analysed applying the Kubelka Munk model [24,25,26].

## 3. Results

### 3.1. *Optical Microscopy and ProX-SEM-EDS* Analysis

**Figure 1:**
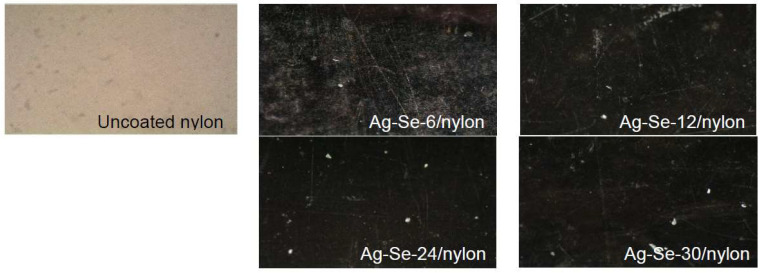
An image of uncoated nylon and obtained Ag–Se/nylon nanocomposites. Magnification 100×.

The optical images of the uncoated nylon and corresponding Ag–Se/nylon nanocomposites are shown in Figure 1. The obtained Ag–Se/nylon nanocomposites were homogeneous, spectacularly reflecting with good adherence.

**Figure 2:**
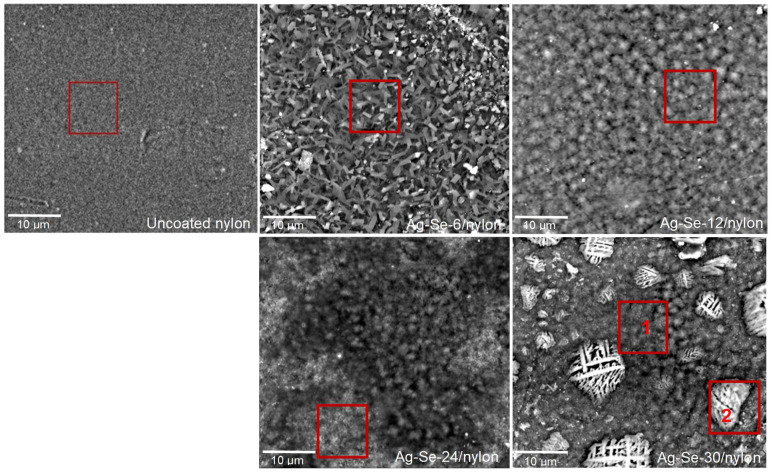
SEM microphotographs of uncoated nylon and Ag–Se/nylon samples with the marked area where the elemental composition was determined by Phenom ProX-SEM-EDS.

The SEM analysis (Figure 2) indicated that the progression of surface morphology changes is significantly dependent on the selenium deposition time. 

As it can be seen, uncoated nylon showed fine dispersion and compact surface morphology. Different small pinholes, bumps, as well as some traces of cracking were visible on the top surface. The surface area of Ag–Se/nylon nanocomposites became larger due to the unevenness and multiple roughnesses, which can be considered as a major source of energy absorption. The surface morphology of Ag–Se-6/nylon sample contained various roads (average size 1.5–4 μm) and irregularly shaped pyramidal-like sub-micron structures (average size 0.5–1 μm). It must also be noted that these units were stacked on top of each other, indicating different stages of growth.

The elemental composition from SEM-EDS analysis of a large area (10 × 11.5 μm^2^) of an Ag–Se-6/nylon sample confirmed the presence of carbon (C), oxygen (O), nitrogen (N), selenium (Se), sulphur (S), and silver (Ag) (Table 1). The ratio of the Ag/Se atomic concentrations *f* was 1.24, and it confirmed the overall deficiency of the silver. By increasing the selenium deposition time up to 12 h, we observed an orderly array of tightly packed spherical-like structures. The average diameter of these spherical-like structures was about 3–4 μm. Detailed analysis of the micrograph shows that these spherical-like structures in fact were clumped in various sized clusters. The dark spots are due to the pits in the surface. A similar surface morphology was also visible in the case of the Ag–Se-24/nylon sample. The different clusters of various spherical particles were formed. In addition, the more heterogeneous surface morphology is evident when compared with the Ag–Se-12/nylon sample. There were deeper (darker) areas, and, above them, there were brighter areas consisting of small >0.5 μm derivatives. As expected, Phenom ProX-SEM-EDS spectra from the Ag–Se-12/nylon and Ag–Se-24/nylon samples (Table 1) confirmed that silver, selenium, and sulphur were present in higher concentrations compared with the values of the Ag–Se-6/nylon sample (Table 1). The ratios of *f* were 1.81 and 1.34 for the Ag–Se-12/nylon and Ag–Se-24/nylon samples, respectively. With a further increase in the selenium deposition time up to 30 h, significant changes in the Ag–Se-30/nylon sample surface morphology could be discerned. The varying size grains, which created different small granular islands, are visible. Various grid-like structures of irregular shapes in the size of 1–15 μm were randomly arranged on the top-surface of these granules. Granular morphology exhibited *f* = 2.03 (Table 1, Ag–Se-30/nylon sample area 1), while, for the grid-like structures, *f* was 2.29 (Table 1, Ag–Se-30/nylon sample area 2). The ProX-SEM-EDS spectra analysis pointed out that not only Ag_2_Se nanoparticles, but also Ag was clearly concentrated on the grid-like structures. Metallic silver is the most likely impurity in the chemical deposition of Ag_2_Se films [5]:

Se_(s)_ + 4AgNO_3(aq)_ + 3H_2_O → 4Ag_(s)_ + H_2_SeO_3(aq)_ + 4HNO_3__(aq)_. (8)

As discussed in ref. [27], excess of Ag may be incorporated in various ways: as point defects, as adsorbed metal chains, or as three-dimensional nano- or microscale in-homogeneities. The silver-rich Ag_2_Se films exhibit the both negative and linear positive magnetoresistance effects [7,28], and no saturating magnetoresistance [7], which predestines them for various applications.

Extraneous elements C and N came from the nylon matrix since it is the largest part of the Ag–Se/nylon samples (Table 1). The higher content of oxygen observed in all the obtained samples as compared with that of the uncoated nylon sample distinctly supports the penetration of oxygen-containing ions, such as SeO_3_^2–^, SO_3_^2–^, or SO_4_^2–^. Although SEM/EDS is useful for the identification of the elemental distribution in micro-domains, however, it must still be appreciated that the elemental information obtained from the micron region is naturally of a heterogeneous nature when compared with bulk analyses.

**Figure 3:**
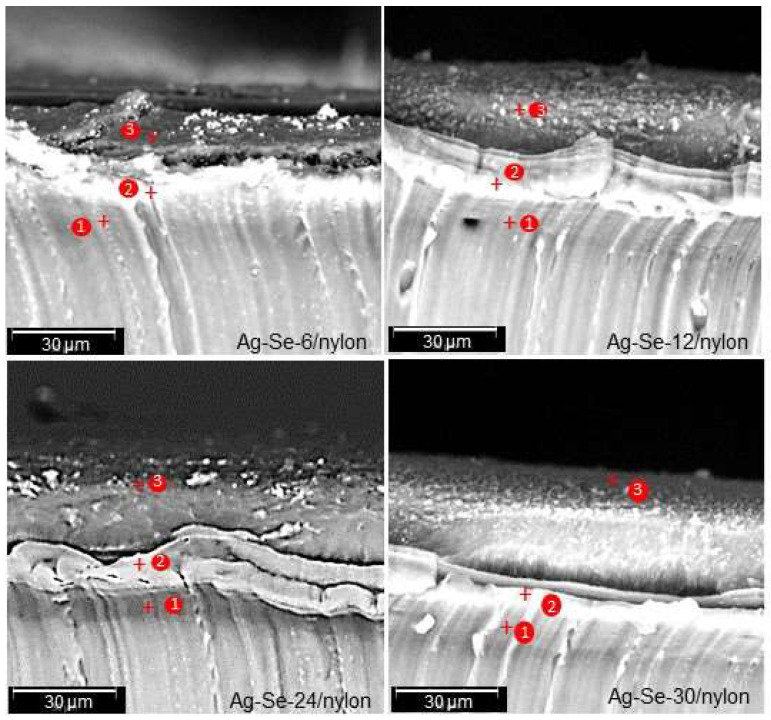
Cross-sectional micrographs of Ag–Se/nylon samples with marked points where the elemental composition was determined by Phenom ProX-SEM-EDS.

As discussed above, hydrophilic nylon absorbs various ions from aqueous electrolyte solutions. To collect the relevant information, the cross-sectional analysis was performed, and the representative results are shown in Figure 3.

Three different layers with varying fractional thickness (Table 2) can be clearly identified from the cross-sectional micrographs of Ag–Se/nylon samples. The boundary between each layer is well defined: a highly dense component depicting a homogeneous diffusion layer (grey), an intermediate layer (white), which seems to be composed of coalescence particles, and, finally, the topmost (dark) layer.

The fractional thickness of the topmost layer increased with the selenium deposition time (Table 2), while the fractional thickness of the intermediate layer decreased. The fractional thickness of the diffusion layer showed a non-monotonic character. The total thickness increased with an increase of the selenium deposition time (Table 2). As an example, it was in the range of 22.83–32.33 and 27.1–39.91 μm for Ag–Se-6/nylon and Ag–Se-30/nylon, respectively. EDS analysis was performed to investigate the chemical composition from each fractional layer of the nanocomposite, and the resulting data (spot 1, spot 2 and spot 3, as marked in Figure 3) are presented in Table 3, Table 4, Table 5 and Table 6, respectively.

The results indicate that the silver, selenium, and sulphur atomic concentration of these fractional layers greatly depends on the selenium deposition time. From the depth profile EDS spectra data (Table 3, Table 4, Table 5 and Table 6), it is clear that sulphur is present in all the three layers, thereby indicating that diffusion occurred during the a–Se/nylon template preparation. Likewise, in the first synthesis step, SeO_3_^2–^, SO_3_^2–^, or SO_4_^2–^ ions as well as selenium nanoparticles penetrate into the polymer matrix until the concentration reaches an equilibrium value. The EDS spectra from the topmost (spot 3), intermediate (spot 2), and diffusion (spot 3) layers indicate non-homogeneous distribution of silver, selenium, and sulphur atomic concentration throughout its thickness. We note that, in all composites, silver was not detected in the diffusion layer (Table 3, Table 4, Table 5 and Table 6). The calculated ratio *f* of the topmost layer was 1.17, 2.02, 1.84, and 2.06 for Ag–Se-6/nylon, Ag–Se-12/nylon, Ag–Se-24/nylon, and Ag–Se-30/nylon, respectively (Table 3, Table 4, Table 5 and Table 6).

### 3.2. XRD Analysis

**Figure 4:**
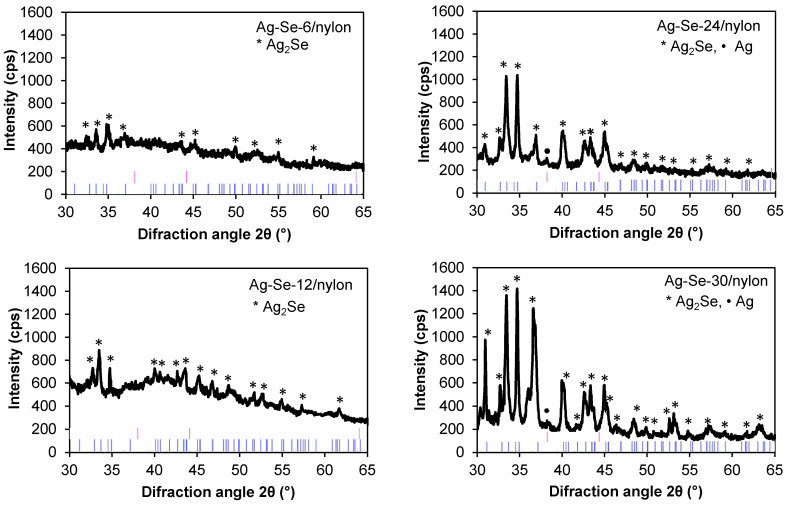
XRD patterns of Ag–Se films on the nylon surface. The black lines are the experimental patterns, and the pink and blue lines label the peaks from Ag (04-003-1472 (Calc., Intensity: 36.0%)) and Ag_2_Se (01-071-2410 (Calc., Intensity: 91.0%)), respectively.

The crystallographic structure of the Ag–Se/nylon nanocomposites was studied by XRD analysis. Our previous XRD studies of the nylon matrix showed that the diffraction pattern features two dominant peaks at 2θ 20.1° and 23.5°, and the peak at 9.4° of lower intensity [29]. As the intensities of nylon XRD peaks sharply exceeded the XRD patterns of the obtained composites, the diffractograms are given in the 30–65° 2θ angular interval. The experimental data were interpreted by using the standard JCPDS cards and the data available in the literature [5,30]. Analysis results are presented in Figure 4.

The Ag–Se-6/nylon nanocomposite showed diffraction peaks corresponding to the orthorhombic naumannite phase of Ag_2_Se (JCPDS # 01-071-2410, λ = 0.15406 nm). As observed from the pattern, the predominant (121) peak of the orthorhombic system represents a preferred orientation along this plane. The identified peak positions coincide well with the ones reported in literature for Ag_2_Se nanowires [5] and nanoparticles [30]. With a further increase in the selenium deposition time (Figure 4 Ag–Se-12/nylon sample), the intensity of the reflection (112) line increased, whereas the number of new peaks corresponding to the silver selenide phase rose. The diffractogram of the Ag–Se-24/nylon nanocomposite showed two sharp lines of nearly equal intensity along (112) and (121) planes, respectively. The orientation along (121) plane became predominant in the Ag–Se-30/nylon nanocomposite. In the Ag–Se-24/nylon and Ag–Se-30/nylon samples diffractograms, the metallic Ag phase (JCPDS # 04-003-1472, λ = 0.15406 nm) was also detected. Usually, the metallic Ag structure is depicted by a sharp XRD peak at 2θ 38.12° corresponding to a preferential (111) texture. Together with the Ag_2_Se phase, a minor amount of elemental selenium may remain not reacted in the deposited film. At room temperature, deposited Se is amorphous and not detected by XRD. Since the Ag_2_SeO_3_, Ag_2_SO_3_, and Ag_2_SO_4_ phases were not found in Ag–Se/nylon nanocomposites, it could be assumed that the SeO_3_^2–^, SO_3_^2–^, and SO_4_^2–^ ions diffused out from the a–Se/nylon template, and reacted with the silver ions to produce these compounds in solution nearby the a–Se/nylon template region. In the other case, the by-products of this reaction, due to high solubility, were removed from sample surface by rinsing with excess hot water before XRD analysis.

### 3.3. AFM Analysis 

**Figure 5:**
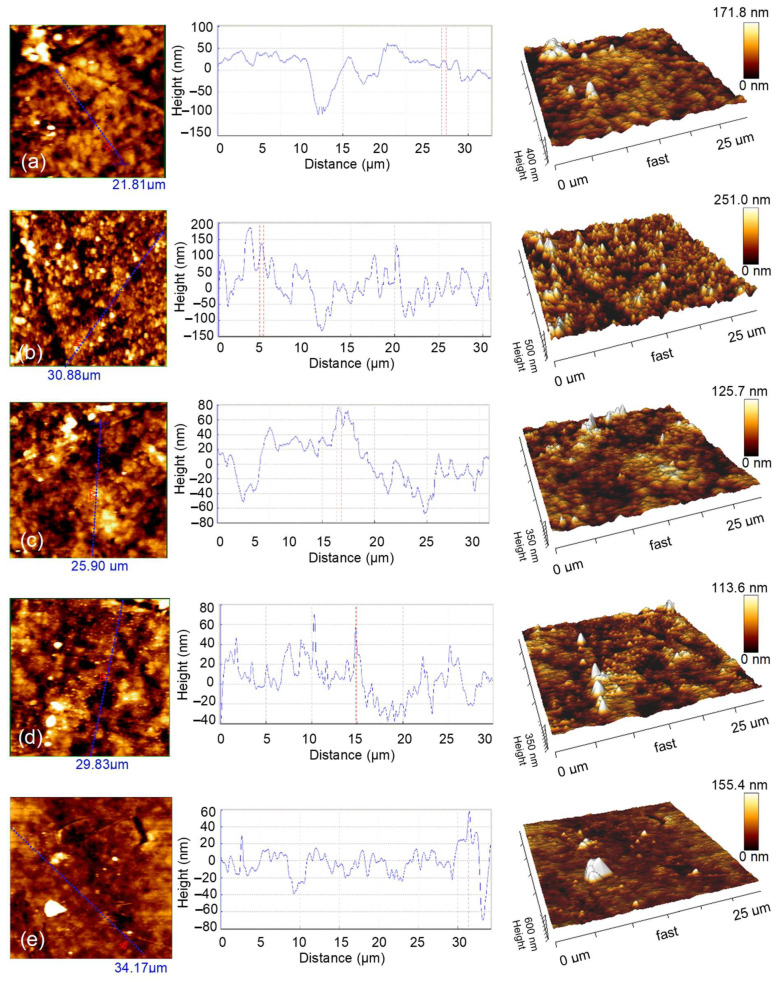
AFM views (left—2D views; center—profile; right—3D views) of (**a**) uncoated nylon, (**b**) Ag–Se-6/nylon, (**c**) Ag–Se-12/nylon, (**d**) Ag–Se-24/nylon, (**e**) Ag–Se-30/nylon.

The 2D and 3D AFM images for scanning areas of 30 × 30 μm^2^ are presented in Figure 5.

The experimental results evidently indicate that the surface topography of the Ag–Se/nylon nanocomposites strongly depends on the selenium deposition time. As it can be seen, the uncoated nylon showed a morphology composed of some bulges, sags, and pores (Figure 5a). These structures of various scales also existed in the Ag–Se-6/nylon sample, and were distributed unevenly in some ranges (Figure 5b). In addition, these features possess different irregular shapes, sizes, and separations. With a further increase in the selenium deposition time (Figure 5c–e), we observed the appearance of particles agglomerates, which form separated islands. There are also various darker areas (spots and channels) filled with the several smaller grains, indicating the non-uniform growth of layers across film thickness. Alternatively, these spots and channels may represent the holes and cracks extracting down to the depth. In addition, randomly distributed pyramidal-like structures were also visible. It is difficult to notice a significant relationship between the size, shape, and the number density of morphological defects and the selenium deposition time. An examination of all AFM images indicates that the smallest number density of morphological defects is in the Ag–Se-30/nylon nanocomposite. The roughness of the composite surface strongly affects the reflectance of light and is crucial for materials application in the optoelectronic devices. The topographical parameters elucidated by using AFM analysis are gathered in Table 7.

**Figure 6:**
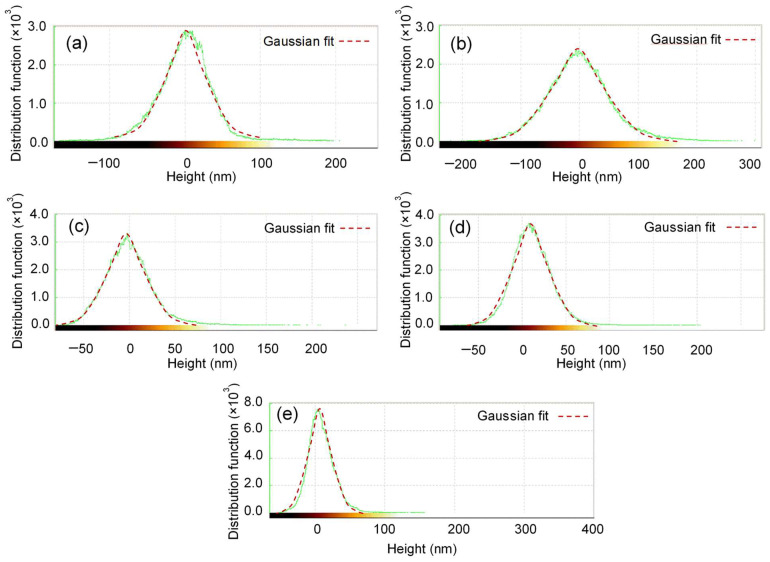
Histogram of the height distribution function in AFM images of (**a**) uncoated nylon, (**b**) Ag–Se-6/nylon, (**c**) Ag–Se-12/nylon, (**d**) Ag–Se-24/nylon, (**e**) Ag–Se-30/nylon samples.

The root-mean-square surface roughness (R_q_) of the obtained composites decreased with an increase of the selenium deposition time from 12 to 24 h, and, in Ag–Se-12/nylon and Ag–Se-24/nylon nanocomposites, it was lower than that of uncoated nylon, but it increased with the prolongation of the selenium deposition time up to 30 h (Table 7). The variation trend in the height and roughness values may be related to the deviation of the films composition from the stoichiometric ratio of Ag and Se (Table 1, Table 3, Table 4, Table 5 and Table 6) and variation in the thickness of the Ag_2_Se film (Table 2). Additionally, the mobility and diffusion of the SO_3_^2–^ and SeO_3_^2–^ ions into the sub-surface space of nylon may enhance or inhibit the grain growth and hence affect the surface morphology (R_q_) and roughness of the deposited films. The (R_q_) obtained values of 25.82–57.04 nm reasonably suggest that the surface morphology is responsible for the relatively high refractive indices of the Ag–Se/nylon nanocomposites (as discussed in the section on UV-Vis analysis). The height distribution function gives the number of times that regions of a constant height occur in the morphology of the film [21]. Figure 6 shows a relatively homogeneous particle height distribution for the Ag–Se/nylon nanocomposites. Kurtosis (R_Ku_) and skewness (R_sk_) parameters equaled 3.1 ± 0.1 and 0.2 ± 0.05, respectively, suggesting a quasi-symmetric Gaussian distribution.

### 3.4. UV-Vis Analysis 

**Figure 7:**
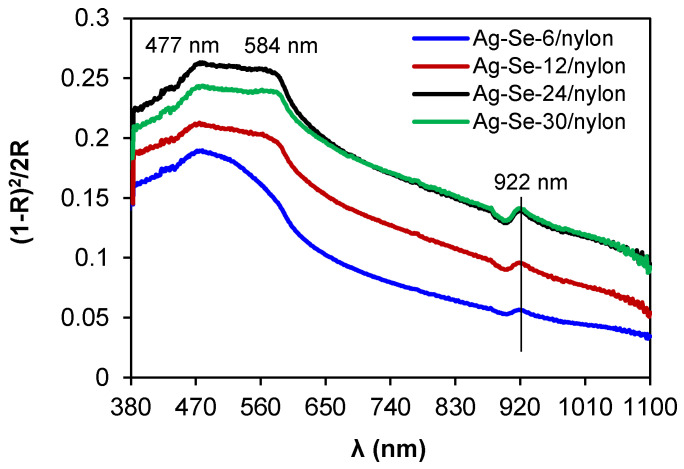
Optical absorption spectra of Ag–Se/nylon nanocomposites.

In the spectrum of the Ag–Se-6/nylon sample (Figure 7), the maximum absorption peak was located at 477 nm, accompanied by a shoulder peak at 584 nm.

It is obvious that both absorption features did not shift with the selenium deposition time, but they were just subjected to an increase in their intensities. The literature reports wide absorption bands in the region between 300 and 600 nm for Ag_2_Se nanoparticles [31]. In the wavelength range higher than 620 nm (near the infrared spectral range), the absorbance monotonically decreased. The peak at 922 nm possibly arises from the absorption by some large coalescing aggregates. The Kubelka–Munk method was applied to determine an optical band gap (*E_op_*). The *E_op_* and the reflectance are interrelated by the equations given below [25,26]: This is example 1 of an equation:

(9) $$F\cdot \left(R\right)=\frac{{\left(1-R\right)}^{2}}{2\cdot R}\;,$$

(10) $$hvF\sim {\left(hv-E_{op}\right)}^{n},$$

where *F*—the Kubelka–Munk function, *R*—the reflectance, *hν*—the photon energy, and *E_op_* is the optical band gap, *n* is a constant characterising the transition mode, *n* = 1/2 or 2 are for the allowed direct or indirect transitions, respectively.

**Figure 8:**
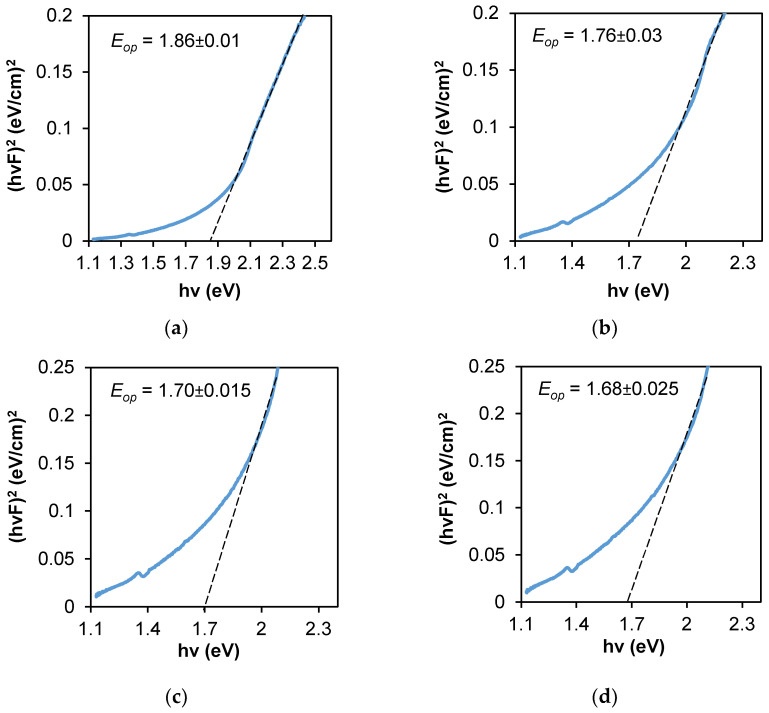
Optical band gap determination from diffuse reflectance spectra by the Kubelka–Munk method: (**a**) Ag–Se-6/nylon; (**b**) Ag–Se-12/nylon; (**c**) Ag–Se-24/nylon; (**d**) Ag–Se-30/nylon samples.

The variation of *(hvF)*^2^ versus *hv* for each composite is shown in Figure 8. The linear part in the higher energy region confirms the allowed direct transition mode. The intersection of a long straight-line part with the photon energy axis depicts *E_op_* value. The obtained values were 1.86 eV, 1.76 eV, 1.70 eV, and 1.68 eV for Ag–Se-6/nylon, Ag–Se-12/nylon, Ag–Se-24/nylon and Ag–Se-30/nylon nanocomposites, respectively. The similar values were reported in ref. [10]. The red shift of *E_op_* values with an increasing of selenium deposition time can be related to the polycrystalline structure of Ag_2_Se and a relatively high surface roughness (Table 7). Additionally, the structural defects generated from the dispersed selenium nanoparticles and adsorbed SeO_3_^2–^, SO_3_^2–^, or SO_4_^2–^ ions in the composites can also contribute to the band gap value [32]. The Ag_2_Se had a narrow band gap (~0.15 eV) in the bulk case at room temperature [33]. The higher *E_op_* values as compared with that of bulk Ag_2_Se suggest that the particle sizes were within the quantum confinement regime [34].

The refractive index difference of constituent materials causes a significant light scattering as well as a loss of transparency in organic-inorganic composites [35]. The following equation was applied to calculate the refractive indices (*n*): [36,37]:

(11) $$n=\frac{-\left(R+1\right)\pm 2\sqrt{R}}{R-1}.$$

**Figure 9:**
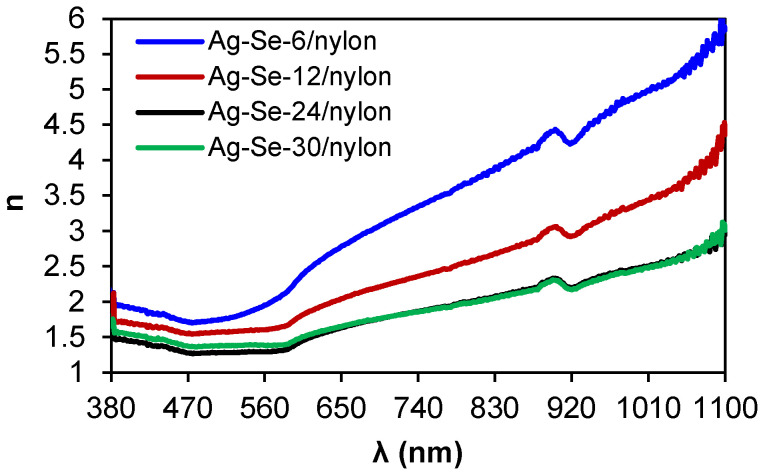
Variation of refractive indices of Ag–Se/nylon nanocomposites on wavelength.

The spectral behaviour of indices (Figure 9) showed a very complex character.

In the spectral region between 380 and 477 nm, the refractive indices of all the investigated Ag–Se/nylon nanocomposites illustrated a slow decrease, which can be explained by a single oscillator model [36]. As we can observe, the refractive indices of the Ag–Se-6/nylon nanocomposite increased in the spectral interval from 477 nm to 584 nm, while, for the three other nanocomposites (Ag–Se-12/nylon, Ag–Se-24/nylon and Ag–Se-30/nylon), it remained nearly constant (the plateau region). In the region at λ > 590 nm, the refractive indices monotonically increased showing an anomalous dispersion [38]. As discussed above, various sub-micron structures, particles and agglomerates covered the nylon surface (Figure 2). These structural elements can also contribute to the anomalous dispersion [39]. At the same time, the refractive indices depend on the selenium deposition time. Specifically, Ag–Se-6/nylon nanocomposite possesses the ultra-high refractive index as compared with other samples (Figure 9). For example, the values of refractive indices for Ag–Se-6/nylon and Ag–Se-30/nylon nanocomposites at λ = 800 nm were 3.68 and 1.98, respectively and coincide well with those reported in ref. [40]. Finally, we must note that the investigated composites possessed high refractive index values. Consequently, they are promising for the development of efficient flat waveguide components and anti-reflective coatings.

## 4. Conclusions

Compact multilayer Ag–Se/nylon semiconductor nanocomposites were synthesised via a template-engaged reaction, which could convert the a–Se layer on nylon into an Ag–Se film. The obtained results imply that the surface morphology, and elemental and phase composition of Ag–Se/nylon nanocomposites as well as their optical properties were highly sensitive to the selenium deposition time on nylon. Scanning electron microscope (SEM) analysis confirmed the development of a very complex surface composed of pyramidal-like sub-micron structures, agglomerates, and grid-like structures. Energy dispersive spectroscopy (EDS) of large areas (10 × 11.5 μm^2^) proved the presence of carbon, oxygen, nitrogen, sulphur, selenium, and silver. The higher content of oxygen was observed in all the obtained composites as compared with that of an uncoated nylon sample, which distinctly suggests the presence of oxygen containing ions, such as SeO_3_^2–^, SO_3_^2–^, or SO_4_^2–^. SEM/EDS cross-sectional analysis proves the multilayer character of the composite with a different individual elemental composition in each layer. X-ray diffraction analysis indicates that Ag–Se/nylon nanocomposites obtained at shorter selenium deposition times exist as polycrystalline naumannite Ag_2_Se; composites obtained at longer selenium deposition times exist as a mixed-phase material composed of Ag_2_Se and metallic Ag. The RMS value obtained from the atomic force microscopy varied from 25.82 nm to 57.04 nm. The direct optical band gap (*E_op_*) was found to be 1.68–1.86 eV. Ag–Se/nylon nanocomposites exhibited high refractive indices in the visible and near infrared region. The presented results are promising for the optimisation of Ag_2_Se/nylon nanocomposite fabrication process, which is one of the most important components in flexible electronics.

## Figures and Tables

**Table 1:** Elemental composition of the Ag–Se/nylon nanocomposites obtained by EDS acquired from the surface area marked in Figure 2.

Sample	Atomic Concentrations, %						
	C	O	N	S	Se	Ag	Ag/Se Concentration Ratio
Uncoated nylon	28.68	44.96	26.36	-	-	-	-
Ag–Se-6/nylon	27.62	52.53	17.31	0.32	0.99	1.23	1.24
Ag–Se-12/nylon	25.14	52.70	17.22	0.90	1.44	2.60	1.81
Ag–Se-24/nylon	29.32	50.05	15.65	1.03	1.69	2.26	1.34
Ag–Se-30/nylon area 1 area 2	27.76 31.51	49.42 44.50	17.40 18.34	0.93 0.81	1.48 1.47	3.01 3.37	2.03 2.29

**Table 2:** Fractional thickness of Ag–Se film layers on nylon.

Layer	Fractional Thickness of the Layers, μm			
	Ag–Se-6/nylon	Ag–Se-12/nylon	Ag–Se-24/nylon	Ag–Se-30/nylon
Topmost (dark)	7.61–9.51	8.67–15.17	8.51–13.70	14.09–19.51
Intermediate (white)	7.61–13.31	8.67–10.14	5.15–7.87	4.34–9.76
Diffusion (grey)	7.61–9.51	8.67–13.00	8.52–11.61	8.67–10.64
Total	22.83–32.33	26.01–38.31	22.18–33.18	27.1–39.91

**Table 3:** Chemical composition from each fractional layer (spot 1, spot 2, and spot 3, as marked in Figure 3) of the Ag–Se-6/nylon nanocomposite obtained by EDS.

Spot	Atomic Concentrations, %						
	C	O	N	S	Se	Ag	Ag/Se Concentration Ratio
1	28.69	44.96	26.35	-	-	-	-
2	34.19	40.10	24.98	0.19	0.54	-	-
3	36.70	37.70	23.30	0.23	0.95	1.12	1.18

**Table 4:** Chemical composition from each fractional layer (spot 1, spot 2, and spot 3, as marked in Figure 3) of the Ag–Se-12/nylon nanocomposite obtained by EDS.

Spot	Atomic Concentrations, %						
	C	O	N	S	Se	Ag	Ag/Se Concentration Ratio
1	20.99	55.98	22.22	0.39	0.42	-	-
2	41.94	37.58	18.68	0.50	0.99	0.31	0.31
3	40.04	36.30	17.80	0.95	3.32	1.59	0.48

**Table 5:** Chemical composition from each fractional layer (spot 1, spot 2, and spot 3, as marked in Figure 3) of the Ag–Se-24/nylon nanocomposite obtained by EDS.

Spot	Atomic Concentrations, %						
	C	O	N	S	Se	Ag	Ag/Se Concentration Ratio
1	27.12	47.98	24.03	0.34	0.53	-	-
2	26.60	45.78	24.47	0.23	0.95	1.97	2.07
3	25.14	52.70	17.22	0.93	1.41	2.60	1.84

**Table 6:** Chemical composition from each fractional layer (spot 1, spot 2, and spot 3, as marked in Figure 3) of the Ag–Se-30/nylon nanocomposite obtained by EDS.

Spot	Atomic Concentrations, %						
	C	O	N	S	Se	Ag	Ag/Se Concentration Ratio
1	30.93	50.91	17.14	0.35	0.67	-	-
2	29.58	45.42	19.06	0.78	1.46	3.70	2.53
3	30.34	46.63	16.53	1.44	1.65	3.41	2.07

**Table 7:** Surface topographical parameters average height (Z_mean_), average roughness (R_a_), root-mean-square surface roughness (R_q_), and peak-to-valley roughness (R_t_) obtained by AFM analysis.

Sample	Z_mean_ (nm)	R_a_ (nm)	R_q_ (nm)	R_t_ (nm)
Uncoated nylon	171.8	28.22	39.06	429.0
Ag–Se-6/nylon	251.0	43.19	57.04	547.0
Ag–Se-12/nylon	125.7	20.67	28.57	350.9
Ag–Se-24/nylon	113.6	18.53	25.82	369.2
Ag–Se-30/nylon	155.4	17.54	35.33	607.6

## Data Availability

Data is contained within the article.
